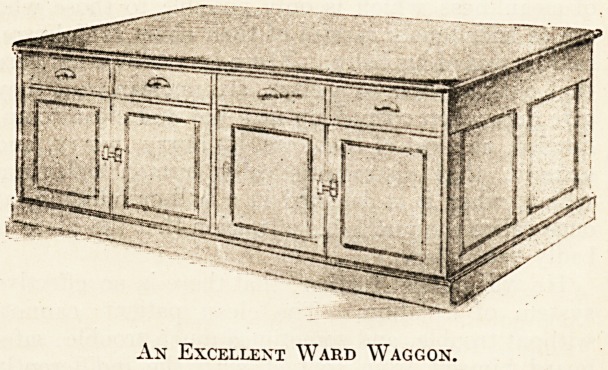# Practical Points

**Published:** 1911-10-07

**Authors:** 


### PRACTICAL POINTS.

### (SUGGESTIONS, WITH PHOTOGRAPHS, AND INQUIRIES ARE INVITED.)

The more hospitals we inspect the more we are impressed with the feeling that a hospital often suffers administratively from a want of knowledge of the best appliances, fittings, furniture, and methods for effective ward decoration. Who but a sister with an artistic mind realises the attractiveness of a comely table at the head of her large ward near the entrance? Indeed, the question of ward tables is a very important one, and most interesting too. We should like to have photographs of the ward tables in use in every hospital where they are made to form a feature or importance is attached to their design or completeness. Probably the ideal ward table from the artistic standpoint is to be found in certain wards of the Gloucestershire Royal Infirmary. We should like to publish a picture of these tables, with their history, which is remarkable, we understand.

By a table we do not mean a ward waggon, though the latter is an invaluable addition to the furniture of a large ward, and we could wish, for the sake of the staff, that its use was more general than it is at present. For the information of those of our readers who have not seen a ward waggon, we give this week an illustration and description of one of the best from every point of view. We shall be very glad to have suggestions for this column, as well as contributions accompanied by drawings or photographs. For the next month the items which have been suggested as of general interest are: (1) Ward tables; (2) dressing waggons, large and small; (3) doctors' portable washstands; (4) patients' lockers. Meanwhile anyone who is in want of information on any special point will, we hope, make use of this column by writing at once to the Editor, as and when occasion may arise.

An Excellent Ward Waggon.

The waggon shown in the illustration is of oak. In the surgical wards the waggon is 6 ft. 6 in. long, 3 ft. broad, and 3 ft. high. It stands flat on the ground without castors. The drawers are 18 in. wide and 5 in. deep. The space below the drawers is divided into two compartments by a central division. In each compartment there is a shelf which can be raised or lowered, as it is on movable fasteners. Both drawers and cupboard go right through the waggon, so that there is access from either side. In the medical wards the waggons are of similar shape to those in the surgical wards, but they are smaller by one drawer and half a cupboard. We are much indebted to the House-Governor of the Royal Victoria Infirmary, Newcastle-on-Tyne, for the above illustration and description.

## Figures and Tables

**Figure f1:**